# Prenatal exposure to perfluoroalkyl and polyfluoroalkyl substances and the risk of hypertensive disorders of pregnancy

**DOI:** 10.1186/s12940-018-0445-3

**Published:** 2019-01-09

**Authors:** Rong Huang, Qian Chen, Lin Zhang, Kai Luo, Lin Chen, Shasha Zhao, Liping Feng, Jun Zhang

**Affiliations:** 1 Ministry of Education-Shanghai Key Laboratory of Children’s Environmental Health, Xinhua Hospital, Shanghai Jiao Tong University School of Medicine, 1665 Kong Jiang Road, Shanghai, 200092 China; 2 Department of Obstetrics and Gynecology, Xin Hua Hospital, Shanghai Jiao Tong University School of Medicine, 1665 Kong Jiang Road, Shanghai, China; 3 School of Public Health, Shanghai Jiao Tong University, 227 S. Chongqing Road, Shanghai, China; 4 Department of Obstetrics and Gynecology, Duke University School of Medicine, Durham, North Carolina 27710 USA

**Keywords:** PFAS, Cord blood, Gestational hypertension, Preeclampsia

## Abstract

**Background:**

Perfluoroalkyl and polyfluoroalkyl substances (PFAS) have been reported to disrupt endocrine system and reproduction. However, epidemiological evidence on the association between PFAS and preeclampsia is inconsistent. We aimed to investigate the association between prenatal PFAS exposure and hypertensive disorders of pregnancy (HDP) in humans.

**Methods:**

PFAS were measured by liquid chromatography system coupled with tandem mass spectrometry in 687 umbilical cord plasma samples collected between 2011 and 2012 in Shanghai, China. Information on HDP including gestational hypertension and preeclampsia was abstracted from medical records. Multiple logistic regression was used to examine the association of each PFAS with gestational hypertension, preeclampsia, and overall HDP in separate models. Elastic net regression with logit link was used to identify independent associations between exposures and outcomes. Logistic regression was used to obtain the unpenalized estimates of the selected PFAS components for the associations with outcomes, adjusting for age, education level, pre-pregnancy BMI, parity, and mutual adjustment of selected PFAS.

**Results:**

The risk of gestational hypertension and preeclampsia was 3.3% and 2.8% in our subjects, respectively. Perfluorobutane sulfonate (PFBS), perfluorohexane sulfonate (PFHxS), perfluoroundecanoic acid (PFUA) were associated with preeclampsia based on elastic net penalty regression. In the fully adjusted statistical model, women with a higher level of standardized ln-transformed PFBS had an increased odds of preeclampsia [adjusted odds ratio (AOR): 1.81, 95% confidence interval (CI): 1.03–3.17], and overall HDP (AOR: 1.64, 95% CI: 1.09–2.47).

**Conclusions:**

Prenatal exposure to PFBS was positively associated with the risk of preeclampsia and overall HDP.

**Electronic supplementary material:**

The online version of this article (10.1186/s12940-018-0445-3) contains supplementary material, which is available to authorized users.

## Introduction

Hypertensive disorders of pregnancy (HDP) are among the most common complications of pregnancy. A national survey involving 112,386 pregnant women from 38 secondary and tertiary hospitals in China in 2011 reported that HDP occurred in 5.2% of pregnancies [1], which is slightly lower than that reported by other international studies [2–4]. HDP are often classified into four categories: 1) chronic hypertension, 2) gestational hypertension, 3) preeclampsia-eclampsia, 4) preeclampsia superimposed on chronic hypertension. Preeclampsia/eclampsia is a more severe form of this disorder and is a major cause of perinatal and maternal morbidity worldwide [5]. It contributes to nearly 10% of stillbirths and 15% of preterm births [6]. It is generally believed that incomplete remodeling of the uterine arteries and insufficient placental perfusion greatly contribute to both gestational hypertension and preeclampsia [7]. However, gestational hypertension seems to be more associated with maternal characteristics than placenta-related factors. For example, it has been reported that women with gestational hypertension are more likely to have higher body mass index than women with preeclampsia [8]. Even though the etiology and pathogenesis of HDP have not been fully elucidated, maternal exposure to environmental pollutants has been considered as an important risk factor of HDP [3].

Perfluoroalkyl and polyfluoroalkyl substances (PFAS) are a large group of manufactured compounds widely used in both industrial and consumer products [9]. Humans are exposed to PFAS through various pathways, including food, water, air, indoor dust and soil [10–13]. Most frequently studied PFAS have a long half-life of 3–5 years in human body [14]. Our previous study found that eight common PFAS were detected in more than 90% of umbilical cord blood samples [15]. Among them, perfluorooctane sulfonate (PFOS) and perfluorooctanoic acid (PFOA) had the highest levels.

Epidemiological studies have provided inconsistent results on the association between PFAS and preeclampsia. Two studies focusing on women who lived in areas with a high PFOA level but with background PFOS level found that PFOA and PFOS were significantly positively associated with preeclampsia with an odds ratio (OR) of 1.1–1.2 across the upper three quintiles for PFOA and OR of 1.13 in relation to a shift from 25th to 75th percentile for PFOS [16, 17], while another study focusing on women with background level of PFAS exposure found that PFOA, PFOS and perfluoroheptane sulfonate (PFHpS) were not associated with preeclampsia, but perfluoroundecanoic acid (PFUA) had an inverse significant association with preeclampsia [HR (95% CI): 0.78 (0.66, 0.92) for per ln-unit] [18]. The inconsistent results may be due to different exposure levels between these two areas. PFAS level in the C8-Health Project took into account the historical PFAS exposure while the study based on Norwegian women did not do so, resulting potential lower estimates of PFAS level due to phase out of PFOS and PFOA in North America and Europe since 2000. The Chinese women probably have a higher level of PFAS exposure than the background exposure level in Norwegian due to continuous production and use of PFAS in China and relocation of PFAS production to China [19]. In addition, a short-chain PFAS, perfluorobutane sulfonate (PFBS) which has been increasingly produced to replace PFOS, has been detected in cord blood samples in the Chinese women [15, 20]. Experimental studies have reported adverse effects of PFBS on the immune and endocrine functions [21, 22]. Therefore, it is possible that these adverse effects may also interfere with the remodeling of uterine spiral arteries, the pivotal feature of normal placentation, and contribute to the development of preeclampsia and gestational hypertension [23, 24]. But whether PFBS is associated with preeclampsia and gestational hypertension is still unknown. Therefore, this study aimed to examine the association of PFAS exposure with preeclampsia and gestational hypertension in the Chinese population.

## Methods

### Study design and participants

The present analysis is a cross-sectional study. From 2011 and 2012, 687 women who had a singleton pregnancy and came for delivery at two large hospitals in Shanghai were recruited. A face-to-face interview was conducted by trained nurses to collect information on maternal age, education level, pre-pregnancy weight, and height. Information on parity and pregnancy-related complications was abstracted from medical records. Cord blood samples were collected shortly after birth. Among the 687 subjects, 13 subjects were excluded because of missing information on maternal age, education level, pre-pregnancy BMI, and parity, resulting in 674 valid subjects. A written consent was obtained from each woman. This study was approved by the Ethics Committees of all involved research institutions and hospitals.

### Outcomes

As noted above, information on chronic hypertension before pregnancy, gestational hypertension, and preeclampsia was obtained from medical records. Gestational hypertension was defined as new onset of hypertension (systolic blood pressure ≥ 140 mmHg or diastolic blood pressure ≥ 90 mmHg) after 20 gestational weeks. Preeclampsia was defined as new onset of hypertension (systolic blood pressure ≥ 140 mmHg or diastolic blood pressure ≥ 90 mmHg) after 20 gestational weeks accompanied by proteinuria (a urine dipstick of at least +). According to the International Society for the Study of Hypertension in Pregnancy, hypertensive disorders of pregnancy (HDP) included gestational hypertension, preeclampsia, chronic hypertension (essential or secondary), or pre-eclampsia superimposed on chronic hypertension [25]. Chronic hypertension before pregnancy and pre-eclampsia superimposed on chronic hypertension were excluded in the current analysis because our study focused on the association between PFAS and pregnancy-related hypertension.

### Blood sampling and exposure assessments

Umbilical cord blood samples were collected at delivery and centrifuged at 4000 rpm for 10 min immediately after collection, with plasma separated and stored at − 80 °C until shipping on dry ice to the laboratory for analysis. Detailed method of measuring PFAS has been described elsewhere [15]. A total of 100 μL was used to measure PFAS for each plasma sample. This method has been cross-validated with a research lab at the Aarhus University in Denmark. PFOSA and PFHpA were not included as they are detected in < 30% samples, PFOS, PFNA, and PFHxS were detected in all the samples, while PFBS, PFOA, PFDA, perfluoroundecanoic acid (PFUA), and perfluorododecanoic acid (PFDoA) had a detection rate above 90%. Those that were not detected were assigned half of the limit of detection (LOD; 0.0045 ng/mL for PFBS, 0.045 ng/mL for PFOA, 0.01 ng/mL for PFDA and PFUA, 0.09 ng/mL for PFOS, 0.03 ng/mL for PFHpA, 0.02 ng/mL for PFNA and PFHxS, 0.12 ng/mL for PFOSA and 0.025 ng/ml for PFDoA). The interassay coefficients of variation (CV) was between 1.7 and 8.4%, and the intra-assay CV was between 0.8 and 8.5% [15].

### Statistical analysis

Considering the multiple correlations between these PFAS, elastic net regression was used to select the exposures that are associated with outcome, while simultaneously accounting for other PFAS exposures. Elastic net regression is a penalized regularization method combined with the properties of ridge and least absolute shrinkage and selection operator (LASSO) penalty [26]. It is well known that in the case of multi-collinearity among examined exposure, ridge penalty shrinks the coefficients of correlated exposures towards each other while LASSO selects one of them and discard the others, avoiding the unstable estimates of correlated exposures in ordinary regression approaches [27]. However, ridge regression retains all predictor variables and cannot produce a parsimonious model, and LASSO regression selects a subset of predictors and owns a poor prediction performance relative to ridge regression if there are high correlations between predictors [26]. By combining ridge and LASSO penalty, elastic net regression does variable selection and continuous shrinkage, and selects groups of correlated exposures as a whole. As such, it has been widely used as a multi-pollutant model that support the identification of the dominant pollutants that are associated with outcome while dealing with multi-collinearity in environmental epidemiological studies [28, 29]. For the elastic net regression with logit link, the turning parameters of ridge and LASSO penalty were selected via 10-fold cross-validation (CV) with the selection criteria of minimum misclassification error. The process was repeated 100 times. After the variable selection, the associations of PFAS with gestational hypertension, preeclampsia, and overall HDP were reassessed by separate multiple logistic regression models.

For regression models, a set of covariates that were selected based on a directed acyclic graph were set as confounders (Additional file 1: Figure S1), including maternal age, educational level, parity and pre-pregnancy BMI (calculated as weight in kilograms divided by height in meters squared). Because only a small proportion of mothers smoke before pregnancy (*N* = 10, proportion = 1.5%) and during pregnancy (*N* = 3, proportion = 0.4%) in our subjects, smoking is unlikely to confound the association assessed in this study and was not included as a confounder. These covariates were included in the elastic net regression without penalization. Before regression analysis, PFAS concentrations were first ln-transformed to mitigate the effects of their right-skewed distribution and then were centered and standardized with one-standard deviation based on ln-transformed scale. Additionally, PFAS concentration was analyzed as both a continuous variable (scaled ln-transformed) and categorical variables in tertiles (T1/T2/T3) with the lowest tertile (T1) being the reference group. *P*-values for linear trend of odds ratio for hypertensive disorders of pregnancy in relation to different level of each PFAS concentration was obtained by treating the categorical PFAS variables as continuous variables in the regression model. Preeclampsia might affect kidney function and alter PFAS concentration, which may be most prominent for PFAS with a short half-life, such as PFBS. To improve the assessment of whether such as effect might have occurred, a sensitivity analysis was conducted for PFBS with further adjustment of birthweight and gestational age. Results were given in Additional file 2: Table S1. Statistical analyses were conducted using RStudio version 1.1.453 (2009–2018 RStudio, Inc) and fit elastic net regression using the glmnet package.

## Results

The risk of gestational hypertension, preeclampsia and overall HDP was 3.3, 2.8, and 6.1%, respectively, in our study. Table 1 shows that the mean age of these women was 29.3 years, with an average pre-pregnancy BMI of 21.3 kg/m^2^. More than 90% of them were nulliparous, and nearly 90% had college education. Women with HDP were a little older [Mean (SD) of HDP vs Normotensive women: 30.6 (4.5) vs 29.2 (3.8), *P* = 0.02], and had a higher pre-pregnancy BMI than normotensive women [Mean (SD) of HDP vs Normotensive women: 23.1 (3.8) vs 21.2 (3.1), *P* < 0.001].

**Table 1:** Basic characteristics of the subjects by hypertensive disorders of pregnancy (HDP) (*n* = 686)

	Total (*n* = 686)	HDP (*n* = 42)	Normotensive (*n* = 644)	^#^*P* values
	Mean (SD) or N (%)			
Age (years)	29.3 (3.8)	30.6 (4.5)	29.2 (3.8)	0.02
Pre-pregnancy BMI (Kg/m^2^)	21.3 (3.2)	23.1 (3.8)	21.2 (3.1)	< 0.001
Parity				
Nulliparous	626 (91.5)	39 (92.9)	587 (91.4)	0.4
Parous	58 (8.5)	3 (7.1)	55 (8.6)	
Education level				
Less than college	95 (13.9)	4 (9.5)	91 (14.1)	
^a^College degree	529 (77.2)	33 (78.6)	496 (77.1)	0.6
Postgraduate degree	61 (8.9)	5 (11.9)	56 (8.7)	

^#^P values were from two-tailed Student’s t tests for continuous variables, and Chi-square tests for categorical variables between HDP and normotensive women

^a^College degree: post-secondary education with 3 or 4 years education in college or university

**Fig. 1:**
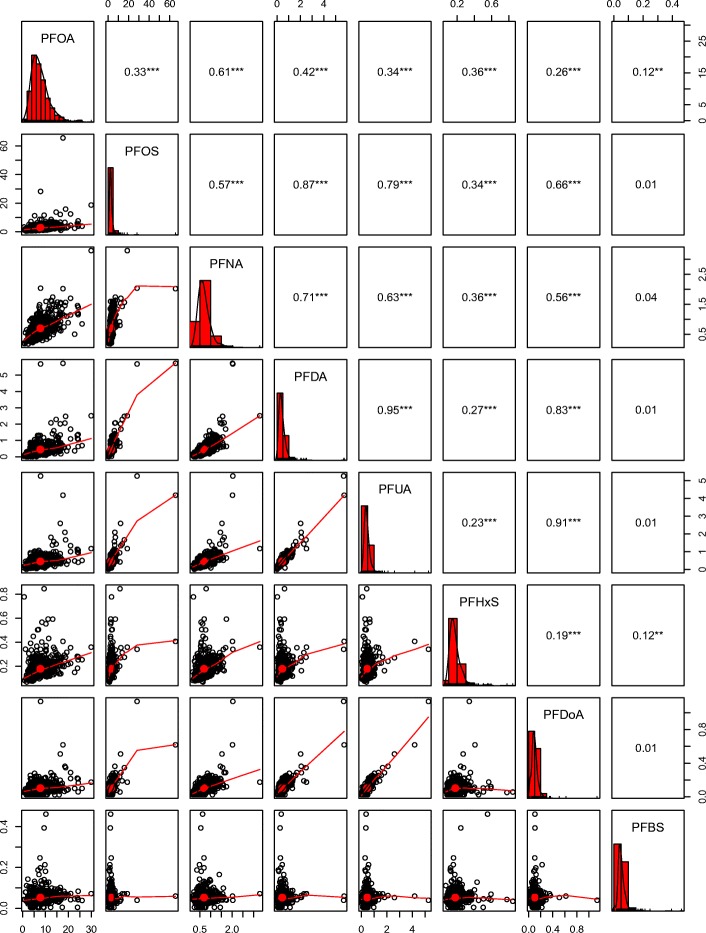
Correlation coefficients among the PFAS. **P < 0.01, *** < *P* < 0.001. Note: Non-straight lines mean that the relation between two PFAS may be non-linear

A total of 8 PFAS components were quantifiable in more than 70% of blood samples (Table 2). PFOA had the highest median concentration (6.98 ng/ml), followed by PFOS (2.38 ng/ml), PFNA (0.64 ng/ml), PFUA (0.40 ng/ml), PFDA (0.36 ng/ml), PFHxS (0.16 ng/ml), PFDoA (0.094 ng/ml) and PFBS (0.047 ng/ml). Figure 1 shows that the Pearson correlation coefficients between the eight PFAS components ranged from 0.01 to 0.95.

**Table 2:** Plasma concentrations of 8 perfluoroalkyl and polyfluoroalkyl substances in our sample

Perfluoroalkyl substances	Abbreviated Name	% > LOD	Plasma concentration (ng/ml) by percentile		
			25th	50th	75th
Perfluorooctanoic acid	PFOA	99.9	4.95	6.98	9.54
Perfluorooctane sulfonate	PFOS	100	1.81	2.38	3.23
Perfluorononanoic acid	PFNA	100	0.50	0.64	0.83
Perfluoroundecanoic acid	PFUA	99.9	0.29	0.40	0.53
Perfluorodecanoic acid	PFDA	99.1	0.23	0.36	0.54
Perfluorohexane sulfonate	PFHxS	100	0.132	0.16	0.20
Perfluorododecanoic acid	PFDoA	90.4	0.069	0.094	0.13
Perfluorobutane sulfonate	PFBS	97.2	0.037	0.047	0.061

LOD (ng/ml): PFOSA (0.12), PFHpA (0.03), PFOS (0.09), PFNA (0.02), PFHxS (0.02)

Table 3 shows odds ratio and corresponding 95% CI of the association between each PFAS and hypertensive disorders of pregnancy after adjusting for potential confounders.

**Table 3:** Odds ratios for hypertensive disorders of pregnancy associated with cord blood concentrations of perfluoroalkyl and polyfluoroalkyl substances

	Hypertensive disorders of pregnancy	Preeclampsia	Gestational hypertension
	^a^OR (95% CI)		
PFOA			
T1	1	1	1
T2	0.85 (0.38–1.88)	2.23 (0.67–7.44)	0.33 (0.10–1.11)
T3	0.96 (0.44–2.10)	1.41 (0.38–5.14)	0.77 (0.30–2.01)
^b^Standardized	1.02 (0.73–1.44)	1.12 (0.68–1.84)	0.95 (0.61–1.48)
PFOS			
T1	1	1	1
T2	0.55 (0.24–1.25)	0.59 (0.19–1.87)	0.54 (0.17–1.66)
T3	0.82 (0.39–1.72)	0.70 (0.23–2.08)	0.95 (0.36–2.49)
^b^Standardized	0.85 (0.62–1.17)	0.83 (0.52–1.32)	0.87 (0.57–1.34)
PFUA			
T1	1	1	1
T2	1.29 (0.62–2.68)	0.89 (0.31–2.52)	1.75 (0.65–4.70)
T3	0.64 (0.27–1.52)	0.51 (0.15–1.76)	0.81 (0.25–2.66)
^b^Standardized	0.82 (0.61–1.10)	0.82 (0.55–1.23)	0.84 (0.58–1.22)
PFDA			
T1	1	1	1
T2	1.23 (0.58–2.59)	1.16 (0.38–3.53)	1.26 (0.48–3.31)
T3	0.78 (0.34–1.80)	1.00 (0.31–3.19)	0.63 (0.20–2.00)
^b^Standardized	0.85 (0.63–1.14)	0.94 (0.60–1.46)	0.79 (0.55–1.15)
PFDoA			
T1	1	1	1
T2	0.92 (0.44–1.93)	1.25 (0.43–3.59)	0.71 (0.26–1.91)
T3	0.53 (0.23–1.22)	0.60 (0.17–2.14)	0.50 (0.17–1.50)
^b^Standardized	0.74 (0.55–1.00)	0.83 (0.54–1.29)	0.70 (0.47–1.03)
PFNA			
T1	1	1	1
T2	0.38 (0.15–0.94)	0.28 (0.06–1.37)	0.47 (0.16–1.41)
T3	0.87 (0.43–1.79)	1.40 (0.51–3.83)	0.56 (0.20–1.41)
^b^Standardized	0.86 (0.62–1.19)	1.10 (0.33–3.71)	0.74 (0.48–1.15)
PFHxS			
T1	1	1	1
T2	0.93 (0.44–1.99)	1.10 (0.36–3.38)	0.83 (0.31–2.22)
T3	0.59 (0.26–1.34)	0.80 (0.25–2.60)	0.48 (0.16–1.43)
^b^Standardized	0.77 (0.54–1.09)	0.81 (0.49–1.33)	0.75 (0.47–1.19)
PFBS			
T1	1	1	1
T2	1.0 (0.40–2.47)	2.08 (0.51–8.50)	0.55 (0.16–1.92)
T3	2.21 (1.00–4.88)	3.41 (0.91–12.7)	1.57 (0.59–4.17)
^b^Standardized	1.53 (1.04–2.25)	1.69 (0.98–2.90)	1.36 (0.82–2.25)

Abbreviations: *T1* tertile 1, *T2* tertile 2, *T3* tertile 3

^a^Adjusting for age, education, pre-pregnancy BMI, and parity

^b^Standardized: PFAS concentration was subtracted by mean and then divided by its standard deviation

Table 4 shows that after adjusting for potential confounders, in the multiple-exposure elastic net regressions, PFBS, PFHxS and PFDoA were selected (beta coefficient from elastic net regression [β_EN_]~ = 0) for the overall HDP, while PFBS, PFHxS and PFUA were selected for preeclampsia, none of the PFAS components was selected for gestational hypertension.

**Table 4:** Multiple-exposure elastic net penalized regression models (β_EN_) for hypertensive disorders of pregnancy

PFAS	Hypertensive disorders of pregnancy	Preeclampsia	Gestational hypertension
PFOA	0		0
PFOS	0		0
PFUA	0	−0.10	0
PFDA	0		0
PFDoA	−0.20		0
PFNA	0		0
PFHxS	−0.13	−0.06	0
PFBS	0.32	0.34	0

Regression coefficients (β_EN_) represent the change in log-odds per increment in standardized ln-transformed PFAS

Table 5 shows that in the unpenalized logistic regression models with adjustment of potential confounders, one unit increase in standardized PFBS concentration was associated with a higher risk of HDP [Adjusted odds ratio (AOR): 1.64, 95% CI: 1.09–2.47], and preeclampsia (AOR: 1.81, 95% CI: 1.03–3.17). PFHxS (AOR: 0.79, 95% CI: 0.55–1.13) and PFDoA (AOR: 0.76, 95% CI: 0.55–1.04) were non-significantly and negatively associated with HDP. PFHxS (AOR: 0.82, 95% CI: 0.49–1.37) and PFUA (AOR: 0.82, 95% CI: 0.53–1.27) were non-significantly and negatively associated with preeclampsia.

**Table 5:** Logistic regression models for the selected exposures and hypertensive disorders of pregnancy/preeclampsia

PFAS	Hypertensive disorders of pregnancy	Preeclampsia
	^a^AOR (95% CI)	
PFBS		
^b^Standardized	1.64 (1.09–2.47)	1.81 (1.03–3.17)
T1 (≤0.0398)	1	1
T2 (0.0399–0.0554)	0.89 (0.39–2.44)	2.09 (0.51–8.53)
T3 (0.0556–0.4612)	2.26 (1.02–5.02)	3.51 (0.94–13.2)
P value for linear trend	0.03	0.05
PFHxS		
^b^Standardized	0.79 (0.55–1.13)	0.82 (0.49–1.37)
T1 (≤0.11402)	1	1
T2 (0.1403–0.1831)	0.94 (0.43–2.03)	1.14 (0.36–3.58)
T3 (0.1834–0.8465)	0.61 (0.26–1.41)	0.92 (0.27–3.11)
P value for linear trend	0.79	0.88
PFDoA		
^b^Standardized	0.76 (0.55–1.04)	
T1 (≤0.0775)	1	NA
T2 (0.0776–0.1118)	0.89 (0.42–1.88)	
T3 (0.112–1.1357)	0.54 (0.23–1.29)	
P value for linear trend	0.77	NA
PFUA		
^b^Standardized	NA	0.82 (0.53–1.27)
T1 (≤0.3276)		1
T2 (0.3277–0.4808)		0.83 (0.29–2.41)
T3 (0.4819–5.2653)		0.49 (0.13–1.75)
P value for linear trend	NA	0.28

Variance inflation variance (VIF) for exposures ranged from 1.01 to 1.1

^a^Adjusting for age, education, pre-pregnancy BMI, parity and mutual adjustment of PFAS including in the corresponding model

Abbreviations: *T1* tertile 1, *T2* tertile 2, *T3* tertile 3

^b^Standardized: PFAS concentration was subtracted by mean and then divided by its standard deviation

Additional file 2: Table S1 shows the results of the association between PFAS and hypertensive disorders of pregnancy with further adjustment of gestational age and birth weight in addition to those adjusted in the model presented in Table 5. Similar results were found.

We also explored the joint effect of the PFAS on hypertensive disorders in pregnancy using the structural equation model (Additional file 3: Figure S2). The odds ratio and corresponding 95% confidence interval (95% CI) of PFAS were 0.99 (0.97, 1.02), 0.98 (0.95, 1.01) and 0.98 (0.94, 1.01) for preeclampsia, gestational hypertension, and hypertensive disorders of pregnancy, respectively.

## Discussion

Our study used elastic net regression models to select a subset of PFAS components most strongly related to HDP and found that PFBS exposure during pregnancy was significantly positively associated with HDP and preeclampsia. However, this study found that preeclampsia was not associated with PFOA and PFOS, which were different from that found in the US-based C8 Health Project which reported both PFOA and PFOS were significantly positively associated with preeclampsia [16, 17]. This study is also different from another study from Norway which found that PFUA had an inverse association with preeclampsia, while PFOS and PFHpS had no association with preeclampsia [18]. The differences between these studies may be due to different measure of PFAS exposure and statistical analysis methods, or different diagnosis methods of preeclampsia.

PFAS was measured in cord blood samples in this study. However, maternal blood samples during mid-pregnancy was used in the study focusing on Norwegian women [18], PFOA was indirectly estimated using environmental, exposure, and pharmacokinetic modeling for each participant [17], and blood samples collected up to 5 years after pregnancy were used to estimate PFAS concentration [16]. Although PFAS in cord blood was used to estimate maternal level in this study, it was reported that PFAS concentration in the cord plasma ranged between 30 to 79% of maternal concentration for PFOA, PFOS, PFHxS, PFUA, PFNA [30], and nearly 100% for PFBS [31].

PFOA in this study was more than two times that reported in the study of Norwegian women [18], which was consistent with another study in Shanghai reporting that maternal serum concentration of PFOA was much higher (mean = 11.6 ng/mL) than that in Norwegian counterparts (1.5 ng/mL) [32]. However, PFOA in this study was slightly lower than that reported in the C8-Health Project, which is consistent with previous two studies in Shanghai [13, 17, 32]. Without adjusting for other PFAS, our study is consistent with the C8-Health Project in that PFOA was positively but non-significantly associated with preeclampsia. However, the study focusing on the Norwegian women did not find such an association. Both this study and the study on the Norwegian women used preeclampsia cases validated by medical records, while self-reported preeclampsia was used in the C8-Health Project [16–18]. Usage of validated preeclampsia case could largely exclude the possibility of misclassification of outcomes.

PFOS in this study was much lower than reported in the other studies [16, 18]. This is partly due to that the efficiency of placental transfer for PFOS is only about 30% [30]. Previous studies have shown that PFOS level of maternal blood samples was similar in Shanghai, Norway, and US [13, 16, 18]. PFOS in cord blood was highly correlated with that in maternal blood (spearman correlation coefficient = 0.74) [30], so the cord blood PFOS level is well representative of the level in maternal blood. Nevertheless, neither PFOA nor PFOS was selected to be associated with preeclampsia in the elastic net regression model which accounts for the correlation between each PFAS.

It has been reported that PFAS in maternal and cord blood samples could be highly correlated, with correlation coefficients ranging from 0.52 to 0.95 [33, 34]. Low correlation might have attenuated the association between PFAS and HDP. The pathogenesis of preeclampsia has not been fully understood, but shallow placentation and endothelial dysfunction may play a key role [35]. It is believed that defective placentation, with shallow trophoblast invasion into the maternal decidual and spiral arteries in early pregnancy, is the starting point in the pathogenesis of preeclampsia [35]. Consequently, placental hypoperfusion and ischemia may lead to changes in the cytokine production and secretion. The imbalance in these cytokines may lead to maternal endothelial dysfunction and subsequently affect cardiovascular system and cause high blood pressure [35].

Despite that PFBS is assumed to be a safe substitute of PFOS, several studies have shown that PFBS has endocrine disruption effects [21, 36], toxicity in human placental trophoblast cells [21] and neuronotypic cells [37], immunotoxicity [22, 38], and transcriptional effects [39]. Some of these toxicities coincide with the pathophysiology of preeclampsia [35], but no previous epidemiological studies have explored the association between prenatal PFBS exposure and HDP.

PFBS may have adverse effects on the immune and endocrine function in human cells, at a concentration level that did not cause cytotoxicity. It has been reported that PFBS interfered with inflammatory cytokines and NF-κB activation, affecting the fine-tuning of pro- and anti-inflammatory microenvironment of the uteroplacental site, and contributing to the dysfunction in the trophoblast activities and vascular endothelial cells function [22–24, 40–42]. The endocrine disrupting effects of PFBS on placental cells have also been demonstrated. Gorrochategui et al. found that PFBS may suppress aromatase activity directly in human placental choriocarcinoma cell line [21], disrupting the regulation of the estrogen levels, which is essential for maintaining healthy pregnancy.

Our study has several limitations. Firstly, PFAS were measured in cord blood rather than in maternal blood. One may question the temporality of the association. One would argue that since preeclampsia occurred before birth, the observed association lacks temporality and, therefore, may not be causal. Indeed, it is possible that preeclampsia might affect liver and kidney functions, leading to less secretion of PFBS and more accumulation in the body, i.e., a reverse causation. However, the sensitivity analysis which further adjusted for birth weight and gestational age produced similar results, indicating that birth weight and gestational age were not associated with PFBS level. Therefore, at least the duration of maternal transfer of PFBS was not affected. On the other hand, maternal-fetal transfer of PFAS has been reported in both human studies and animal studies [20, 30, 43–45]. Our own recent research found that the median ratio of PFBS concentration in cord serum to maternal serum was nearly 1 among 369 paired samples of maternal blood and cord blood [31]. The extremely high transmission through the placenta might be due to the short carbon-chain of PFBS [30]. Thus, the cord blood level of PFBS can represent maternal blood level well. However, PFBS level in late pregnancy may not necessarily represent that in early pregnancy. Previous studies have reported that PFOA and PFOS decreased across pregnancy due to increased glomerular filtration rate (GFR) and subsequent increased elimination speed by urine [43, 46–48]. Therefore, PFAS level in multiple time points across pregnancy is desired to better reflect the exposure level. Additionally, the magnitude of the association assessed in this study may be biased if the association between PFAS and hypertensive disorders of pregnancy is not linear. Secondly, the relatively small sample size in this study provided limited statistical power and resulted in wide 95% CIs. However, the direction and magnitude of the associations in this study are unlikely to be substantially biased.

## Conclusion

Plasma PFBS in cord blood was positively associated with preeclampsia in a dose-response pattern. Further large-scale prospective studies in human and animal experiments are warranted to examine the relationship between PFBS exposure and preeclampsia, and to elucidate the underlying biological mechanisms.

## Additional files

Additional file 1: **Figure S1**. The directed acyclic graph of the association between each PFAS and hypertensive disorders of pregnancy. (PNG 48 kb)

Additional file 2: **Table S1**. Logistic regression models for the selected exposures and hypertensive disorders of pregnancy/preeclampsia. (DOCX 15 kb)

Additional file 3: **Figure S2**. Structural equation model including a joint latent PFAS concentration. The latent PFAS concentration is manifested by the observed PFOA, PFOS, PFHxS, PFNA, PFDoA, PFDA, PFUA, and PFBS. “Confounders” are age, education level, parity, and pre-pregnancy BMI. (PNG 29 kb)
