# Supplementary figures and images for: Prenatal exposure to perfluoroalkyl and polyfluoroalkyl substances and the risk of hypertensive disorders of pregnancy
Source: Environ Health. 2019 Jan 9;18:5. doi: 10.1186/s12940-018-0445-3 (PMC6327470; doi:10.1186/s12940-018-0445-3)

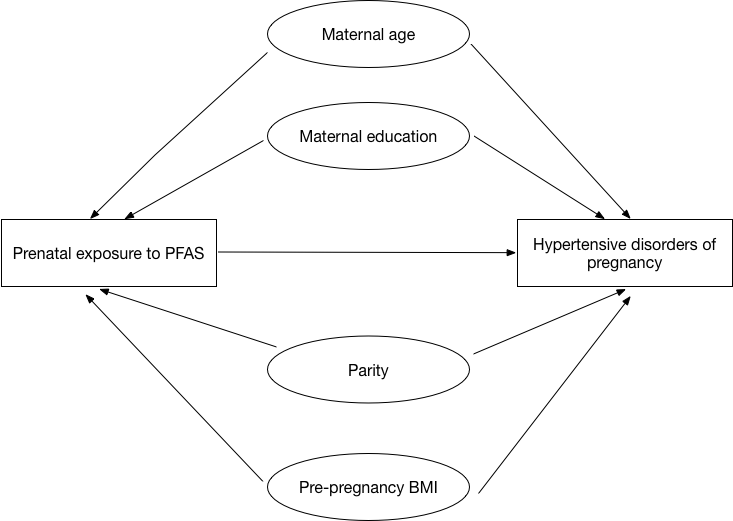

Supplement: Supplementary file 1 — Figure S1. The directed acyclic graph of the association between each PFAS and hypertensive disorders of pregnancy. (PNG 48 kb) [file 12940_2018_445_MOESM1_ESM.png]

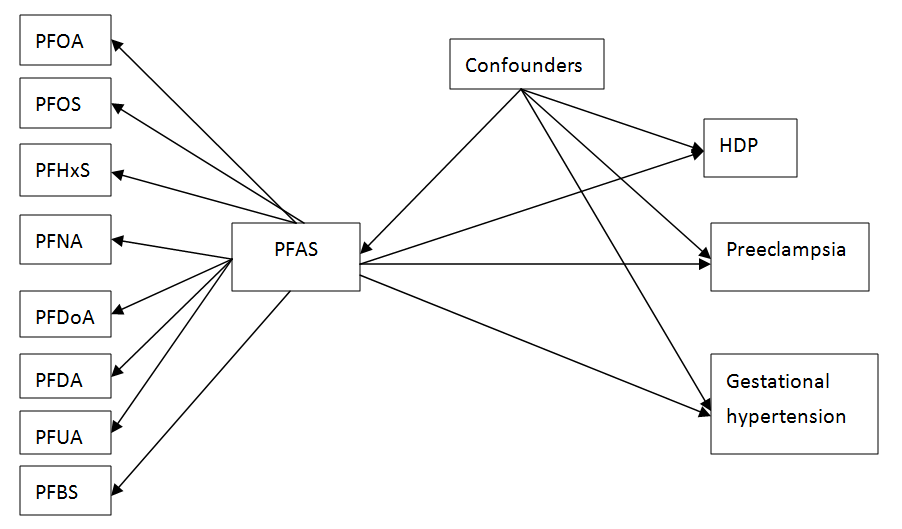

Supplement: Supplementary file 3 — Figure S2. Structural equation model including a joint latent PFAS concentration. The latent PFAS concentration is manifested by the observed PFOA, PFOS, PFHxS, PFNA, PFDoA, PFDA, PFUA, and PFBS. “Confounders” are age, education level, parity, and pre-pregnancy BMI. (PNG 29 kb) [file 12940_2018_445_MOESM3_ESM.png]
