# Supplementary material for: Prenatal exposure to perfluoroalkyl and polyfluoroalkyl substances and the risk of hypertensive disorders of pregnancy
Source: Environ Health. 2019 Jan 9;18:5. doi: 10.1186/s12940-018-0445-3 (PMC6327470; doi:10.1186/s12940-018-0445-3)
Supplement: Supplementary file 2 — Table S1. Logistic regression models for the selected exposures and hypertensive disorders of pregnancy/preeclampsia. (DOCX 15 kb) [file 12940_2018_445_MOESM2_ESM.docx]

Table S1. Logistic regression models for the selected exposures and hypertensive disorders of pregnancy/preeclampsia

| PFAS | Hypertensive disorders of pregnancy | Preeclampsia |
| --- | --- | --- |
|  | ^#^AOR (95% CI) |  |
| PFBS  *Standardized  T1 (≤0.0398)  T2 (0.0399-0.0554)  T3 (0.0556-0.4612) | 1.71 (1.13-2.60)  1  1.06 (0.42-2.70)  2.57 (1.13-5.87) | 1.98 (1.11-3.54)  1  2.84 (0.62-13.1)  5.17 (1.18-22.6) |
| P value for linear trend | 0.015 | 0.02 |
| PFHxS  *Standardized  T1 (≤0.11402)  T2 (0.1403-0.1831)  T3 (0.1834-0.8465) | 0.83 (0.59-1.18)  1  0.99 (0.50-2.16)  0.69 (0.29-1.61) | 0.87 (0.53-1.43)  1  1.16 (0.36-3.70)  1.07 (0.30-3.74) |
| P value for linear trend | 0.39 | 0.91 |
| PFDoA  *Standardized  T1 (≤0.0775)  T2 (0.0776-0.1118)  T3 (0.112-1.1357) | 0.76 (0.55-2.60)  1  0.81 (0.38-1.75)  0.56 (0.24-1.35) | NA |
| P value for linear trend | 0.24 | NA |
| PFUA  *Standardized  T1 (≤0.3276)  T2 (0.3277-0.4808)  T3 (0.4819-5.2653) | NA | 0.86 (0.54-1.34)  1  0.83 (0.28-2.47)  0.58 (0.15-2.16) |
| P value for linear trend | NA | 0.41 |

^#^Adjusting for age, education, pre-pregnancy BMI, parity, gestational weeks, birth weight, and mutual adjustment of PFAS included in the corresponding model.

Abbreviations: T1, tertile 1; T2, tertile 2; T3, tertile 3.

*Standardized: PFAS concentration was subtracted by mean and then divided by its standard deviation.
